# Angiotensin 1–7 in an experimental septic shock model

**DOI:** 10.1186/s13054-023-04396-8

**Published:** 2023-03-13

**Authors:** Bruno Garcia, Fuhong Su, Francesca Manicone, Laurence Dewachter, Raphaël Favory, Amina Khaldi, Alexander Moiroux-Sahroui, Anthony Moreau, Antoine Herpain, Jean-Louis Vincent, Jacques Creteur, Fabio Silvio Taccone, Filippo Annoni

**Affiliations:** 1 Experimental Laboratory of Intensive Care, Université Libre de Bruxelles, Brussels, Belgium; 2 Department of Intensive Care, Erasme University Hospital, Université Libre de Bruxelles, Brussels, Belgium; 3 Laboratory of Physiology and Pharmacology, Université Libre de Bruxelles, Brussels, Belgium; 4 Department of Intensive Care, Centre Hospitalier Universitaire de Lille, Lille, France

**Keywords:** Circulatory failure, Vasopressors, Inflammation, Renin-angiotensin system, Angiotensin (1–7)

## Abstract

**Background:**

Alterations in the renin–angiotensin system have been implicated in the pathophysiology of septic shock. In particular, angiotensin 1–7 (Ang-(1–7)), an anti-inflammatory heptapeptide, has been hypothesized to have beneficial effects. The aim of the present study was to test the effects of Ang-(1–7) infusion on the development and severity of septic shock.

**Methods:**

This randomized, open-label, controlled study was performed in 14 anesthetized and mechanically ventilated sheep. Immediately after sepsis induction by bacterial peritonitis, animals received either Ang-(1–7) (*n* = 7) or placebo (*n* = 7) intravenously. Fluid resuscitation, antimicrobial therapy, and peritoneal lavage were initiated 4 h after sepsis induction. Norepinephrine administration was titrated to maintain mean arterial pressure (MAP) between 65 and 75 mmHg.

**Results:**

There were no differences in baseline characteristics between groups. Septic shock was prevented in 6 of the 7 animals in the Ang-(1–7) group at the end of the 24-h period. Fluid balance and MAP were similar in the two groups; however, MAP was achieved with a mean norepinephrine dose of 0.4 μg/kg/min in the Ang-(1–7) group compared to 4.3 μg/kg/min in the control group. Heart rate and cardiac output index were lower in the Ang (1–7) than in the control group, as were plasma interleukin-6 levels, and creatinine levels. Platelet count and PaO_2_/FiO_2_ ratio were higher in the Ang-(1–7) group. Mean arterial lactate at the end of the experiment was 1.6 mmol/L in the Ang-(1–7) group compared to 7.4 mmol/L in the control group.

**Conclusions:**

In this experimental septic shock model, early Ang-(1–7) infusion prevented the development of septic shock, reduced norepinephrine requirements, limited interleukine-6 increase and prevented renal dysfunction.

**Supplementary Information:**

The online version contains supplementary material available at 10.1186/s13054-023-04396-8.

## Background

Septic shock is defined as life-threatening organ-dysfunction due to a dysregulated host response to infection [1]. The management of septic shock is based on early screening and diagnosis, source control, antibiotic therapy and hemodynamic management, including fluids and vasopressors [2]. Management of septic shock remains challenging, and worldwide mortality related to sepsis remains high [3–5].

Advances in the understanding of the pathophysiology of septic shock have recently involved the renin–angiotensin system (RAS) [6–8]. Angiotensinogen, produced by the liver, is converted into angiotensin I (Ang I) under the activity of renin; Ang I is then cleaved by the angiotensin converting enzyme (ACE) into angiotensin II (Ang II), the main effector of the classical RAS pathway. Ang II exerts its effects mainly through binding to the Ang II-receptor type 1 (AT-1R), inducing a pro-inflammatory response, vasoconstriction, as well as sodium and water retention [9, 10].

In septic shock, the renin concentration and the Ang I/Ang II ratio are elevated and associated with worse outcomes [6, 7]. This elevation could be explained by a decrease in ACE activity, due to endothelial dysfunction [6, 7], or by the rapid degradation of Ang II due to endogenous peptidases, such as dipeptidyl peptidase 3 (DPP-3) [11]. Ang II deficiency could be responsible for an inadequate AT-1R stimulation that could participate in the pathophysiology of vasodilatory shock [8], and Ang II supplementation has proven useful as a second line vasopressor [12]. Nevertheless, the counterbalancing axis of Ang II, mediated by the ACE2/Ang-(1–7)/Mas receptor axis, could also be beneficial for its anti-inflammatory properties [13]. Ang-(1–7) is produced from Ang-II through the action of the peptidase ACE2, or through an intermediate transformation of Ang-I into Angiotensin-(1–9) by ACE2 before its final conversion to Ang-(1–7) by ACE and can bind its specific receptor (Mas R). This leads to a wide range of effects that counteract the pro-inflammatory, pro-apoptotic, pro-fibrotic and vasoconstrictive effects of the AT-1R stimulation induced by Ang-II [14].

Recent studies have shown beneficial effects of administration of Ang-(1–7) in experimental sepsis [15, 16]. Organ dysfunction, and mortality were improved by Ang-(1–7) administration in a cecal ligation and puncture (CLP) model of sepsis in rats [17]. Ang-(1–7) has been also shown to attenuate acute kidney injury in sepsis induced by lipopolysaccharide (LPS), by modulating nuclear factor-kappa B (NF-κB) signaling [15], and to reduce inflammatory cellular infiltrate in a model of experimental acute respiratory distress syndrome (ARDS) [16]. Moreover, circulating Ang-(1–7) concentrations were increased in severe COVID-19 patients, but whether this represents an adaptive response to severe infection has not yet been clarified [18, 19].

We therefore tested the hypothesis that early treatment with Ang-(1–7) in an experimental large animal model would limit the development of septic shock and decrease organ dysfunction.

## Methods

### Study setting

The study followed the EU Directive (2010/63/EU) for animal experiments and was approved by the local animal ethics committee (Protocol number 772N, Comité Ethique du Bien-Être Animal, from the Université Libre de Bruxelles (ULB) in Brussels, Belgium). Experiments were performed in the Experimental Laboratory of Intensive Care of the ULB (LA1230406). The ARRIVE guidelines and MQTiPSS recommendations for translational research in sepsis were followed [20, 21].

An ovine model of fecal peritonitis, adapted from previous experiments [22–24], was used, with 14 domestic female adult (6–8 months) Suffolk sheep included. Only females were used to facilitate access to bladder catheterization and increase homogeneity.

### Experimental procedure

On the day of the experiment, the animals were weighed, premedicated with an intramuscular mixture of 0.25 mg/kg midazolam and 20 mg/kg ketamine, and placed in the supine position. An 18G peripheral cannula was inserted into the cephalic vein to ensure vascular access.

After administration of an intravenous bolus of 30 μg/kg of fentanyl citrate, 1 mg/kg of propofol, and 0.1 mg/kg of rocuronium bromide, an 8 mm endotracheal tube was introduced. All the animals were sedated with 1.8–2.4% alveolar concentration of sevoflurane, and a continuous intravenous infusion of morphine at a rate of 0.2–0.4 mg/kg/h. The optimal dose was determined through repeated pain tests, and in the absence of other explanatory factors, additional boluses of 0.1 mg/kg were given. Rocuronium bromide was administered at 0.1 mg/kg/h for muscle paralysis. Hypoglycemia was avoided by giving a continuous infusion of a 20% glucose solution. A 60 cm long plastic tube (inner diameter 1.8 cm) was inserted via the esophagus into the rumen to drain its content and to prevent rumen distension.

Mechanical ventilation was started in a volume-controlled mode (Primus, Dräger, Lübeck, Germany) using a tidal volume of 8 mL/kg, positive end-expiratory pressure of 5 cmH_2_O, a fraction of inspired oxygen of 30%, ratio of inspiratory time to expiratory time of 1:2 and a square-wave pattern. Respiratory rate was adjusted to maintain end-tidal carbon dioxide pressure (PetCO_2_) between 35 and 45 mmHg. Animals were under mechanical ventilation until the end of the experiment.

A 4.5 G arterial catheter was introduced into the left femoral artery under ultrasound guidance (Vivid E90, GE Machines, USA) connected to a pressure transducer and zeroed at the mid-thorax level. Pulse pressure variation (PPV) was automatically calculated from the arterial femoral signal using the formula [PPV = PP_max_ − PP_min_/(PP_max_ + PP_min_)/2], with PP being the pulse pressure (i.e., the difference between systolic and diastolic arterial pressures), continuously displayed (SC9000, Siemens, Munich, Germany), and exported to a recording station (Notocord-Hem 4.4, Notocord, France). In addition, an 8 Fr introducer was inserted into the left jugular vein, to introduce a 7.5F Swan-Ganz catheter (CCO, Edwards LifeSciences, Irvine, California, USA) into the pulmonary artery. A three-lumen central line catheter was inserted in the right jugular vein to provide fluids and drug infusion. A 14 Fr Foley catheter was inserted into the bladder and connected to a manometer to monitor intra-vesical pressure and to a urine collection bag for monitoring of urine output.

A midline laparotomy was performed. After cecotomy, 1.5 g/kg body weight of feces was collected and stored. The cecum was then closed and repositioned in the abdominal cavity. Two plastic tubes were left behind for later introduction of the feces and peritoneal lavage. After abdominal surgery, the animals were placed prone. Baseline measurements were taken, and feces were then injected into the abdominal cavity.

**Fig. 1:**
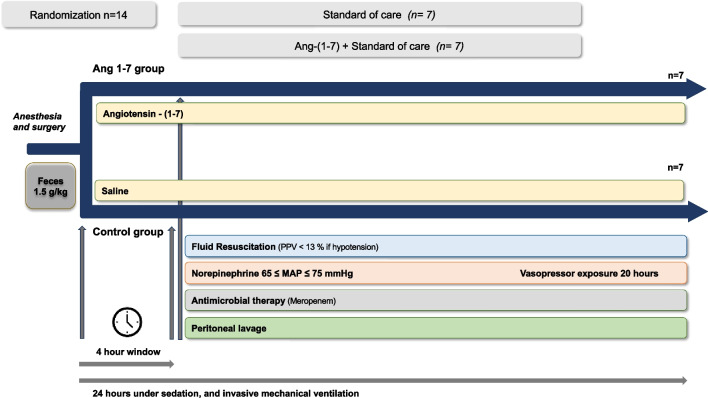
Protocol timeline

Immediately thereafter, seven of the animals received a continuous infusion of 10 μg/kg/h of Ang-(1–7) (Chemcube, Bochum, Germany—Ang-(1–7) group), and seven received a corresponding volume of saline solution (placebo) until the end of the experiment (Fig. 1). The selected dose of Ang-(1–7) was derived from a study using a rat model of ARDS [16], in which a low dose of 0.27 μg/kg/h Ang-(1–7) improved oxygenation and a high dose of 60 μg/kg/h reduced inflammation; an intermediate dose was therefore selected in this experiment.

During the first 4 h, fluids were maintained at 2 mL/kg/h. Then, fluid resuscitation was then started with equal amounts of crystalloid (Plasmalyte, Baxter, USA) and colloid (Geloplasma, Fresenius Kabi, France) solutions, targeting a PPV < 13% in case of MAP ≤ 65 mmHg. Peritoneal lavage was performed using four liters of warm (38° Celsius) saline through the abdominal drain tubes. Intravenous norepinephrine was started if the mean arterial pressure (MAP) was ≤ 65 mmHg despite fluid administration, and titrated to a maximum dose of 5 μg/kg/min.

Four hours after injection of the feces, meropenem was administered as an intravenous bolus of 20 mg/kg, followed by a continuous infusion of 2.5 mg/kg/h until the end of the experiment. Experiments were continued until spontaneous death or for 24 h at which point the animals were euthanatized under deep anesthesia with a bolus injection of 40 mL of potassium chloride solution.

### Data collection and blood sampling

Variables, including MAP (mmHg), pulmonary artery (PA) pressure (mmHg), right atrial pressure (mmHg) and PA wedge pressure (mmHg), were continuously displayed (SC9000, Siemens, Munich, Germany) and exported to an A/D recording station (Notocord-Hem 4.4, Notocord, France). Variables were referenced to the mid-chest level and obtained at end expiration. Core temperature (°C) and cardiac output (L/min) (Vigilance II; Edwards Lifesciences, California, USA), as well as minute volume (mL), plateau pressure (mmHg), expiratory tidal volume (mL), and end-tidal carbon dioxide pressure (mmHg) were continuously monitored. Cardiac index (L/min/m^2^), stroke volume index (mL/m^2^), systemic vascular resistance (dynes/s/cm^5^), and pulmonary vascular resistance (dynes/s/cm^5^) were calculated using standard formulas.

Urine output (UO) was monitored hourly. Arterial and mixed central venous blood gas samples were obtained every hour. Additional arterial samples were obtained at baseline and T4, T8, T12, T16, T20 and T24 hours after sepsis induction for later determination of blood creatinine and interleukin (IL)-6 levels. They were sampled in EDTA-syringes and centrifuged at 3000 rounds per minute for 15 min, then immediately frozen at − 20 °C until analysis.

### Multiplex cytokine magnetic bead panel assay

Protein levels of IL-6 and IL-10 in systemic arterial plasma were determined using a cytokine magnetic bead panel assay (MILLIPLEX® Ovine Cytokine Multiplex Assay, Merck, Germany), according to manufacturer’s instructions. Cytokine concentrations were obtained by referring to a standard curve realized in parallel. Results represented the mean value of two separate measurements performed in duplicate at each time point.

### Statistical analysis

The number of animals was selected based on our previous experience with this animal model [22, 24]. Statistical analysis was performed using Prism 9 (Version 9.1.2 (225). San Diego, CA, USA). Continuous variables are presented as means ± standard deviation (SD) or median [25, 75% interquartile range (IQR)]. To estimate the effect of Ang-(1–7) administration during the observational period, a mixed-effects model with Greenhouse–Geisser correction was used. The effects of time and group, as well as the interaction between group and time, were tested as fixed effects, and animals were introduced as random effects. If there were significant differences, the two-stage linear procedure of Benjamini, Krieger, and Yekutieli, with individual variances, was used for comparison of the means of these variables between the groups at each time point. Differences in time to develop several predefined organ failure parameters and survival time between groups were tested using a log-rank test. A *p* value of < 0.05 was considered statistically significant.

## Results

**Fig. 2:**
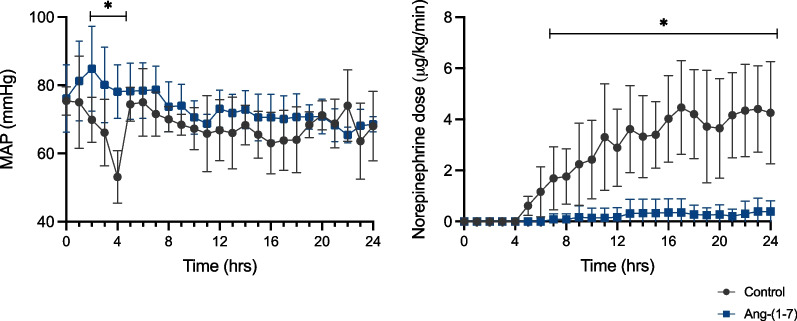
Mean arterial pressure and norepinephrine requirements to maintain MAP between 65 and 75 mmHg during the study period. Values are expressed as mean ± SD

There were no differences between the groups in any baseline values (Additional file 1: Table S1). Body temperature increased more in the control group during the study period than in the Ang-(1–7) group (Additional file 1: Fig. S1). MAP decreased rapidly after feces injection in the control group, with a mean value of 53 ± 8 mmHg 4 h after feces injection, but animals in the Ang-(1–7) group did not develop hypotension (Fig. 2). Norepinephrine requirements were significantly lower in the Ang-(1–7) group than in the control group (Fig. 2). Two animals from the Ang-(1–7) group did not require norepinephrine throughout the whole experiment.

**Fig. 3:**
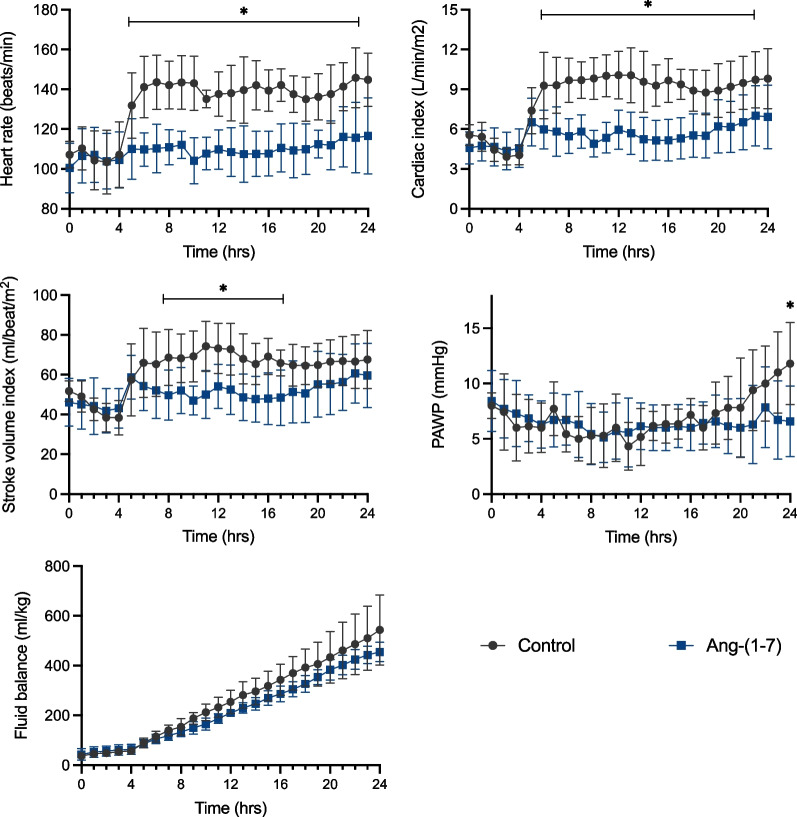
Hemodynamic variables and fluid balance over the study period. *PAWP* Pulmonary artery wedge pressure. Values are expressed as mean ± SD

There were no differences in MAP after fluid resuscitation (Fig. 2). However, heart rate, cardiac index, and stroke volume index were significantly lower in the Ang-(1–7) than in the control group, immediately after the resuscitation process started (Fig. 3). The pulmonary artery wedge pressure (PAWP) remained stable in the Ang-(1–7) group throughout the experiment but increased over the final hours in the control group (*p* = 0.03 vs Ang-(1–7) group at 24 h, Fig. 3). Fluid balance (Fig. 3, Additional file 1: Table S5) and intra-vesical pressure (9 ± 3 in the Ang-(1–7) group vs. 8 ± 4 mmHg in the control group at the end of the experiment, Additional file 1: Table S2) were not significantly different over time in the two groups.

### Lactate, oxygenation and organ dysfunction

**Fig. 4:**
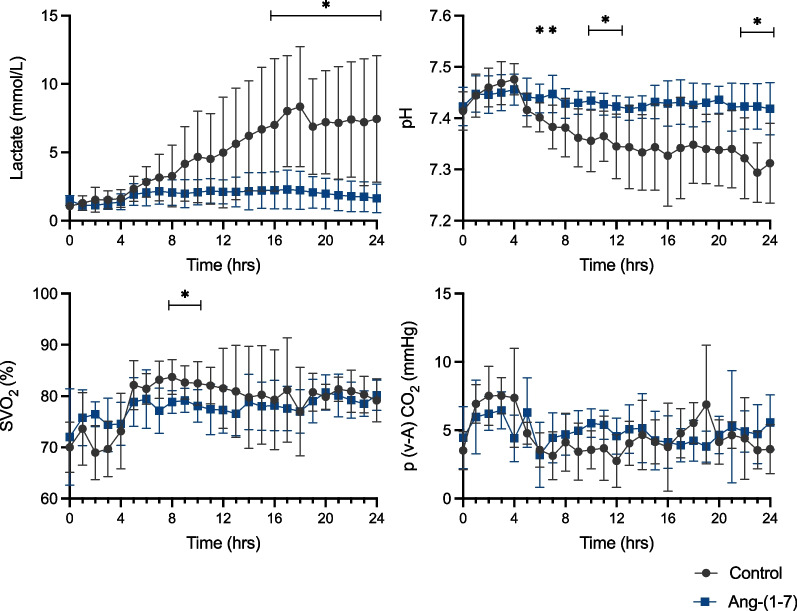
Arterial blood lactate, arterial pH, and oxygenation indices over the study period. Values are expressed as mean ± standard deviation

**Fig. 5:**
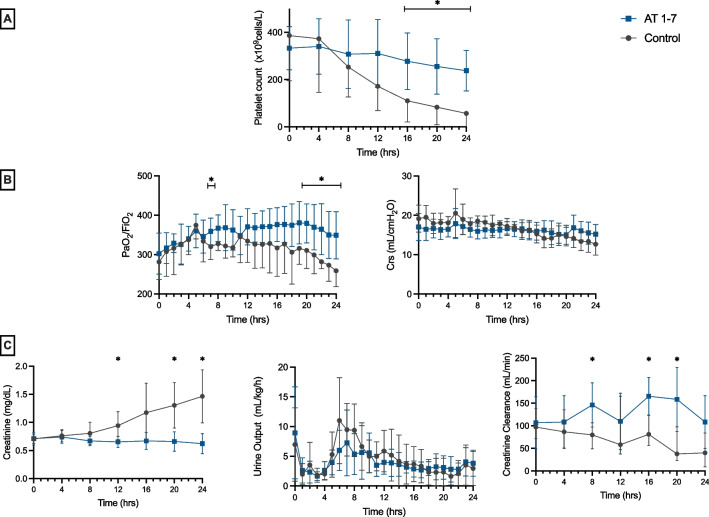
**A** Platelets count, **B** PaO_2_/FiO_2_ ratio, and respiratory system compliance, (Crs= compliance of the respiratory system), **C** Creatinine levels, urine output, and creatinine clearance. Values are expressed as mean ± standard deviation

Arterial lactate levels remained within normal values in 6 of the 7 animals in the Ang-(1–7) group at the end of the experiment but increased in all the animals in the control group (Fig. 4, Additional file 1: Table S3). Arterial pH was significantly higher in the Ang-(1–7) group than in the control group (Fig. 4). No differences were observed in SvO_2_ and P(v-A) CO_2_ (Fig. 4) values between groups. The PaO_2_/FiO_2_ ratio (from 8 h), but not the respiratory system compliance, was significantly higher in the Ang-(1–7) group. Platelet count (from 10 h) was significantly higher in the Ang-(1–7) than in the control group (Fig. 5).

Creatinine concentrations and creatinine clearance were significantly lower in the Ang-(1–7) group, but not urine output (Fig. 5). No differences were observed in white blood cell count, hemoglobin concentration, prothrombin time, activated partial thromboplastin time, or fibrinogen levels (Additional file 1: Table S4).

### Systemic inflammatory cytokines

**Fig. 6:**
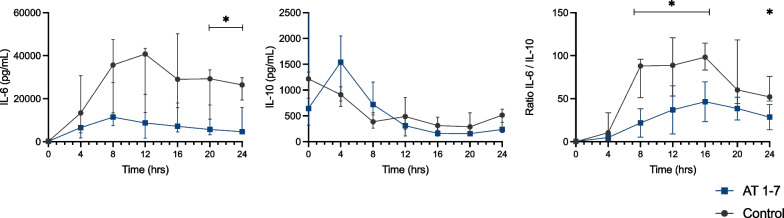
Inflammatory cytokines (IL-6, IL-10 and IL-6/IL-10 ratio) levels at the different timepoints in the two groups. Values are expressed as median and interquartile range

Plasma IL-6 levels increased less in the Ang-(1–7) than in the control group and were significantly lower at the 20- and 24-h time points. There were no differences in IL- 10 levels over time. The IL-6/IL-10 ratio was therefore lower in the Ang-(1–7) group compared to the control group (Fig. 6).

### Survival

All the animals in the Ang-(1–7) group and 5/7 (71%) in the control group were alive at 24 h (*p* = 0.14). Animals died at T12 and T18 in the control group.

## Discussion

In this large animal septic shock model, early Ang-(1–7) administration prevented development of septic shock, as shown by lower norepinephrine requirements, and no increase in arterial lactate levels. Exposure to Ang-(1–7) was also associated with better PaO_2_/FiO_2_ ratio, thrombopenia, and less renal dysfunction.

The benefits of early Ang-(1–7) administration were likely partially related to reduce systemic inflammation. Indeed, Ang-(1–7) administration was associated with less pronounced hyperthermia and a smaller increase in IL-6 levels. Reduced systemic inflammation might explain the less severe vascular dysfunction, with vasoplegia and high norepinephrine requirements to maintain MAP observed only in the control group. These results are consistent with a CLP study in rats, in which Ang-(1–7) administered 3 h after CLP reduced the decrease in MAP and reduced plasma IL-6 levels [17]. However, that experiment was performed in un-resuscitated animals, i.e., without fluid administration, vasopressors or antibiotic therapy, limiting its external validity and human translational ability.

The mechanism behind the preserved vascular tone with Ang-(1–7) may be related to its effect of blocking IL-6, chemokine, and nitric oxide production [25]. Ang-(1–7) is theoretically associated with a vasodilatory effect [14]. However, consistent with what has been reported in the literature in critically ill patients with COVID-19 [26], Ang-(1–7) infusion was not associated with systemic or pulmonary vasodilatory effects in our study, and no hemodynamic safety concerns were observed. Ang-(1–7) has been also described to have potential vasoconstrictive effects, mediated by central vasopressin release or by an interaction of Mas R with AT-1R, especially at high concentration [14], an effect that could participate to the hemodynamic response observed. However, no vasoconstrictive effects have been reported in recent studies [17, 25, 26]. A potential beneficial effect in this model could be related to an improvement in endothelial dysfunction, as *Mas* R stimulation is associated with improved vascular function [14].

Cardiac output and heart rate were higher in the control group than in the Ang-(1–7) group; this could be explained by direct beta-adrenergic stimulation from the higher doses of norepinephrine administered in the control group. This higher stimulation can lead to a downregulation of β-adrenergic receptors and impaired contractility, which is suggested as part of the pathophysiology of septic cardiomyopathy [27, 28]. Ang-(1–7) could be beneficial in reducing norepinephrine exposure and its adverse effects. It may also have direct cardioprotective effects through the ACE2 axis, as hypothesized in heart failure studies [29, 30]. However, we did not specifically assess cardiac function so are unable to comment further on this issue.

The administration of Ang-(1–7) also resulted in less renal dysfunction. First, creatinine levels and creatine clearance were preserved as shown in the Ang-(1–7) group. This is consistent with other pre-clinical studies: Zhu et al. showed, in an LPS model of sepsis, that Ang-(1–7) reduced the levels of urea, creatinine, and cystatin C, with a similar reduction in the inflammatory cytokines tumor necrosis factor (TNF)-α, IL-1, and IL-6 in serum and kidney [15]. There was also a decrease in phosphorylated NF-κB p65 levels in the kidney. No effect was observed on urine output, which remained similar between groups. These results might suggest that the renal effects of Ang-(1–7) are related to the modulation of inflammation.

Ang-(1–7) was also associated with a beneficial effect on gas exchange but not in respiratory system compliance, similarly to a study in ARDS, in which Ang-(1–7) was showed to reduce pulmonary cellular infiltrate and fibrosis [16]. Gerard et al. showed that ACE2 was upregulated in lung tissue and serum during ARDS, with an increase in circulating Ang-(1–7), due to a reproducible response of the lung to acute injury [31]. This potential treatment is currently being tested in a clinical trial in COVID-19-related ARDS patients (NCT04332666).

The immunopathology of sepsis is more complex than an imbalance in pro- and anti-inflammatory responses. In particular, Ang-(1–7) has been showed to reduce apoptosis after LPS administration [25], a mechanism that reduces the host’s repertoire of effector immune cells, and is involved in sepsis-associated mortality [32]. The use of norepinephrine as a vasopressor agent, recently reported to be associated with immunomodulatory effects, may also have modified the immune response in our model [33]. The complexity of the immune effects associated with the RAS during sepsis remains poorly defined; for example, Ang II administration has been showed to increase bacterial clearance and pro-inflammatory response through the AT-1R pathway on myeloid cells [34]. Further studies are needed to understand the complexity of classical and alternative RAS pathways during sepsis, and the mechanisms behind a potential therapeutic effect of RAS modulation.

Two animals died in the control group compared to none in the Ang-(1–7) group. Although our study was underpowered to detect a difference in mortality rate, this finding is consistent with results from the study by Tsai et al. in a rat model of sepsis [17] and may be attributed to reduced organ damage [16].

Our study has several strengths: this large animal model of peritonitis-induced septic shock fulfills preclinical research recommendations for clinical relevance and external validity regarding management, with fluid therapy, source control and vasopressor administration [21]. We used a fluid protocol based on a dynamic parameter of fluid responsiveness, with an objective of PPV < 13% when hypotension occurred [35]. Intra-vesical pressure was monitored to prevent any interaction of increased intra-abdominal pressure on hemodynamic management [36]. Source control, with peritoneal lavage and broad-spectrum antibiotic therapy, was performed [37]. All the catheters were introduced percutaneously under ultrasound guidance, which limits the tissue inflammation related to surgery.

The study also has several limitations: first, the timing of the administration of Ang-(1–7) differs from clinical practice, when treatments are often delayed. However, due to the theoretical vasodilatory effects of Ang-(1–7), we chose to start the treatment at sepsis induction to analyze the effects of high dose of Ang-(1–7) on hemodynamics and to maximize the efficacy. Also, in our experimental model, MAP is usually low when resuscitation starts, and the administration of an agent with possible vasodilatory effects was considered inappropriate. However, we did not observe any safety concerns. Ischemia–reperfusion observed in the control group at T4 could have been responsible for confounding effects, and a later administration of Ang-(1–7) would have been interesting to address this issue. Furthermore, we included young healthy animals, which is different from patients who usually present several comorbidities. We did not perform a dose finding protocol and whether there is a dose-dependent relationship remains to be elucidated. Finally, the best timing for an Ang-(1–7) intervention may be different to that chosen, and potential adverse events related to the modulation of the immune response should be assessed over a longer observation period.

## Conclusions

In a clinically relevant ovine septic shock model, early Ang-(1–7) infusion prevented the development of septic shock, reduced norepinephrine requirements, limited IL-6 increase and reduced renal dysfunction.

## Supplementary Information

**Additional file 1. Table S1.** Baseline variables. **Figure S1.** Core body temperature in the two groups. **Table S2.** Intra vesical pressure in the two groups at the different timepoints. **Table S3.** Arterial lactate in the two groups at the different timepoints. **Table S4.** Blood parameters in the two groups at the different timepoints. **Table S5.** Fluids administration between T4 and T5.

## Data Availability

The datasets used during the current study are available from the corresponding author on reasonable request.
