# Supplementary material for: Angiotensin 1–7 in an experimental septic shock model
Source: Crit Care. 2023 Mar 13;27:106. doi: 10.1186/s13054-023-04396-8 (PMC10010236; doi:10.1186/s13054-023-04396-8)
Supplement: Supplementary file 1 — Additional file 1. Table S1. Baseline variables. Figure S1. Core body temperature in the two groups. Table S2. Intra vesical pressure in the two groups at the different timepoints. Table S3. Arterial lactate in the two groups at the different timepoints. Table S4. Blood parameters in the two groups at the different timepoints. Table S5. Fluids administration between T4 and T5. [file 13054_2023_4396_MOESM1_ESM.docx]

**SUPPLEMENT**

**ANGIOTENSIN 1-7 IN AN EXPERIMENTAL SEPTIC SHOCK MODEL**

Bruno GARCIA^1,2,4^ Fuhong SU^1^ ; Francesca MANICONE^1^ ; Laurence DEWACHTER^3^ ; Raphaël FAVORY^4^ ; Amina KHALDI^1,2^ ; Alexander MOIROUX-SAHROUI^1^ ; Anthony MOREAU^1,2^ ; Antoine HERPAIN^1,2^ ; Jean-Louis VINCENT^2^ ; Jacques CRETEUR^2^ ; Fabio Silvio TACCONE^1,2^ ; Filippo ANNONI^1,2^

^1^Experimental Laboratory of Intensive Care, Université Libre de Bruxelles, Brussels, Belgium

^2^Department of Intensive Care, Erasme University Hospital, Université Libre de Bruxelles, Brussels, Belgium

^3^Laboratory of Physiology and Pharmacology, Université Libre de Bruxelles, Brussels, Belgium

^4^Department of Intensive care, Centre Hospitalier Universitaire de Lille, Lille, France

**Corresponding author**: Bruno GARCIA, MD, MSc, Experimental Laboratory of Intensive Care, Université Libre de Bruxelles, Brussels, Belgium. E-mail: [br.garcia@icloud.com](mailto:br.garcia@icloud.com)

**TABLE S1: BASELINE VARIABLES**

| Variables | Control group  (n = 7) | ANG-(1-7) group  (n = 7) |
| --- | --- | --- |
| Body weight  (kg) | 35.9 ± 0.7 | 34 ± 2.1 |
| Temperature  (°C) | 38.2 ± 0.8 | 38.1 ± 0.7 |
| Heart rate  (beats/min) | 107 ± 7 | 100 ± 12 |
| Cardiac index  (L/min/m^2^) | 5.5 ± 0.8 | 4.6 ± 1.2 |
| Stroke volume  (mL) | 52 ± 5 | 46 ± 12 |
| Stroke volume index  (mL/kg) | 1.33 ± 0.2 | 1.19 ± 0.3 |
| MAP  (mmHg) | 75 ± 4 | 76 ± 10 |
| MPAP  (mmHg) | 20 ± 7 | 20 ± 4 |
| PAWP  (mmHg) | 8 ± 1 | 8 ± 3 |
| SvO_2_  (%) | 70 ± 5 | 72 ± 9 |
| P (a-V) CO_2_  (mmHg) | 4 ± 1 | 4 ± 2 |
| Lactate  (mmol/L) | 1.1 ± 0.2 | 1.5 ± 0.4 |
| PaO_2_/FiO_2_  (mmHg/%) | 281 ± 45 | 302 ± 52 |
| Creatinine  (mg/dL) | 0.7 ± 0.1 | 0.7 ± 0.1 |

MAP: Mean arterial pressure; MPAP: mean pulmonary arterial pressure; PAWP: PAWP: pulmonary artery wedge pressure, SvO_2_: mixed venous oxygen saturation; P (a-V) CO_2_: partial arterio-venous CO_2_ difference

**FIGURE S1: Core body temperature in the two groups**

**TABLE S2**

**Intra vesical pressure in the two groups at the different timepoints**

|  | Group | BL | T4 | T8 | T12 | T16 | T20 | T24 |
| --- | --- | --- | --- | --- | --- | --- | --- | --- |
| Intra-vesical pressure  (mmHg) | *Ang-(1-7)*  *Control* | 1 ± 1  1 ± 1 | 2 ± 2  5 ± 2 | 5 ± 4  7 ± 2 | 7 ± 4  7 ± 2 | 8 ± 4  8 ± 3 | 7 ± 4  8 ± 2 | 8 ± 4  9 ± 3 |

**TABLE S3**

**Arterial lactate in the two groups at the different timepoints**

|  | Group | BL | T4 | T8 | T12 | T16 | T20 | T24 |  |
| --- | --- | --- | --- | --- | --- | --- | --- | --- | --- |
| Lactate  mmol/L | ***Ang-(1-7) n°1*** | **1** | **1.5** | **2** | **1.9** | **1.5** | **1.5** | **1.1** |  |
|  | ***Ang-(1-7) n°2*** | **1.3** | **1.7** | **2** | **1.8** | **1.8** | **2.1** | **1.7** |  |
|  | ***Ang-(1-7) n°3*** | **1.2** | **0.9** | **1.1** | **1.3** | **1.4** | **1.2** | **1** |  |
|  | ***Ang-(1-7) n°4*** | **1.5** | **1** | **1.3** | **1.3** | **1** | **0.9** | **0.9** |  |
|  | ***Ang-(1-7) n°5*** | **2.1** | **2.5** | **4** | **3.9** | **5.1** | **4.3** | **3.9** |  |
|  | ***Ang-(1-7) n°6*** | **1.5** | **0.9** | **1.9** | **2.3** | **2.1** | **1.9** | **1.3** |  |
|  | ***Ang-(1-7) n°7*** | **1.8** | **1.2** | **2.1** | **2.2** | **2.6** | **2** | **1.5** |  |
|  | ***Control***  ***n°1*** | **1.4** | **2.8** | **3.8** | **8.8** | **Death** |  |  |  |
|  | ***Control***  ***n°2*** | **1.4** | **1.4** | **2** | **3.2** | **6.3** | **10.4** | **11.1** |  |
|  | ***Control***  ***n°3*** | **0.9** | **1.1** | **1.5** | **1.7** | **3** | **3.5** | **2.9** |  |
|  | ***Control***  ***n°4*** | **1** | **1.3** | **1.8** | **4.2** | **7.9** | **8.3** | **7.1** |  |
|  | ***Control***  ***n°5*** | **1** | **2.1** | **7.6** | **12.8** | **15.1** | **Death** |  |  |
|  | ***Control***  ***n°6*** | **0.9** | **1.7** | **3.2** | **5.5** | **8.5** | **10.9** | **13.1** |  |
|  | ***Control***  ***n°7*** | **0.9** | **0.9** | **1.8** | **2.5** | **2.7** | **2.9** | **3** |  |

BL: baseline

**TABLE S4**

**Blood parameters in the two groups at the different timepoints**

|  | Group | BL | T4 | T8 | T12 | T16 | T20 | T24 |
| --- | --- | --- | --- | --- | --- | --- | --- | --- |
| WBC  (/mm^3^) | ***Ang-(1-7)***  ***Control*** | 5924 ± 2374  4181 ± 2283 | 3749 ± 1604  2824 ± 1722 | 2227 ± 1191  1989 ± 953 | 1969 ± 687  1966 ± 996 | 2281 ± 693  3008 ± 712 | 2821 ± 1068  3678 ± 1069 | 3001 ± 1190  4048 ± 1014 |
| Hb  (g/dL) | ***Ang-(1-7)***  ***Control*** | 7.2 ± 1.3  6.8 ± 1 | 9.1 ± 1.7  9.7 ± 1.6 | 8.8 ± 1.3  9.2 ± 1.3 | 8.4 ± 1  9.1 ± 1.4 | 8.5 ± 0.9  9.5 ± 1.9 | 8.4 ± 1  8.7 ± 1.1 | 8.7 ± 1.3  8 ± 1 |
| PT  (sec) | ***Ang-(1-7)***  ***Control*** | 15 ± 2  14 ± 1 | 15 ± 1  15 ± 2 | 19 ± 3  19 ±2 | 25 ± 5  34 ± 10 | 25 ± 5  35 ± 8 | 29 ± 5  35 ± 8 | 28 ± 7  36 ±12 |
| aPTT  (sec) | ***Ang-(1-7)***  ***Control*** | 28 ± 13  34 ± 5 | 33 ±11  37 ±14 | 53 ± 34  45 ± 2 | 48 ±14  63 ±30 | 48 ±20  67 ±13 | 64 ± 28  88 ± 28 | 62 ± 33  74 ± 31 |
| Fibrinogen  (g/L) | ***Ang-(1-7)***  ***Control*** | 0.9 ± 0.2  1.2 ± 0.2 | 1 ± 0.1  1 ±0.2 | 0.7 ± 0.2  0.7 ± 0.1 | 0.7 ± 0.2  0.6 ± 0.3 | 0.5 ±0.1  0.4 ±0.1 | 0.4 ± 0.1  0.3±0.1 | 0.5±0.2  0.5±0.2 |
| AST  (UI/L) | ***Ang-(1-7)***  ***Control*** | 247 ± 190  106 ± 16 | 255 ± 169  133 ± 14 | 199 ± 147  104 ± 24 | 167 ± 123  105 ± 33 | 144 ± 99  122 ± 36 | 129 ± 100  125 ± 28 | 175 ± 111  138 ± 37 |
| ALT  (UI/L) | ***Ang-(1-7)***  ***Control*** | 18 ± 6  13 ± 2 | 22 ± 10  15 ± 2 | 16 ± 8  11 ± 4 | 16 ± 9  11 ± 4 | 16 ± 10  14 ± 6 | 17 ± 12  13 ± 5 | 19 ± 9  14 ± 6 |

WBC: white blood count; Hb: hemoglobin; PT: thrombin time; aPTT: activated partial thromboplastin time; AST: aspartate aminotransferase; ALT: alanine aminotransferase

**TABLE S5: Fluids administration between T4 and T5**

|  | **Control group** | | | **Ang (1-7) group** | | |
| --- | --- | --- | --- | --- | --- | --- |
| **Animals** | **Fluids (mL)** | **Weight**  **(Kg)** | **mL/kg** | **Fluids (mL)** | **Weight**  **(Kg)** | **mL/kg** |
| **1** | 1080 | 34 | 32 | 800 | 33 | 24 |
| **2** | 840 | 36 | 23 | 640 | 35 | 18 |
| **3** | 880 | 37 | 24 | 980 | 38 | 26 |
| **4** | 1360 | 35 | 39 | 820 | 34 | 24 |
| **5** | 1720 | 37 | 46 | 700 | 32 | 22 |
| **6** | 1520 | 35 | 43 | 1440 | 34 | 42 |
| **7** | 1350 | 35 | 39 | 340 | 32 | 11 |
| **Mean** | **1250±329** | **36±1.1** | **35±9** | **817±339** | **34±2** | **24±10** |
